# Expression of human *Cfdp1* gene in *Drosophila* reveals new insights into the function of the evolutionarily conserved BCNT protein family

**DOI:** 10.1038/srep25511

**Published:** 2016-05-06

**Authors:** Giovanni Messina, Maria Teresa Atterrato, Laura Fanti, Ennio Giordano, Patrizio Dimitri

**Affiliations:** 1 Istituto Pasteur Fondazione Cenci-Bolognetti, Laboratorio di Genomica Funzionale e Proteomica di Sistemi modello, Dipartimento di Biologia e Biotecnologie, Sapienza Università di Roma, Roma, Italy; 2 Dipartimento di Biologia, Università Federico II, Napoli, Italy

## Abstract

The Bucentaur (BCNT) protein family is widely distributed in eukaryotes and is characterized by a highly conserved C-terminal domain. This family was identified two decades ago in ruminants, but its role(s) remained largely unknown. Investigating cellular functions and mechanism of action of BCNT proteins is challenging, because they have been implicated in human craniofacial development. Recently, we found that YETI, the *D. melanogaster* BCNT, is a chromatin factor that participates to H2A.V deposition. Here we report the effects of *in vivo* expression of CFDP1, the human BCNT protein, in *Drosophila melanogaster*. We show that CFDP1, similarly to YETI, binds to chromatin and its expression results in a wide range of abnormalities highly reminiscent of those observed in *Yeti* null mutants. This indicates that CFDP1 expressed in flies behaves in a dominant negative fashion disrupting the YETI function. Moreover, GST pull-down provides evidence indicating that 1) both YETI and CFDP1 undergo homodimerization and 2) YETI and CFDP1 physically interact each other by forming inactive heterodimers that would trigger the observed dominant-negative effect. Overall, our findings highlight unanticipated evidences suggesting that homodimerization mediated by the BCNT domain is integral to the chromatin functions of BCNT proteins.

The human craniofacial development protein 1 (CFDP1) is a 299 amino acid long polypeptide which belongs to the evolutionarily conserved family of Bucentaur (BCNT) proteins (Fig. 1) characterized by a highly conserved C- terminal BCNT domain^1^,^2^. Despite its widespread distribution in animals and plants, the function(s) and mechanism(s) of action of BCNT proteins need to be clarified.

Defects of craniofacial development represent the main cause of infant mortality and disability in humans, but the genetic causes and underlying developmental etiology remain largely unknown. Several lines of evidence are suggestive for an involvement of *Cfdp1* gene orthologs in craniofacial development of vertebrates. First, human *Cfdp1* gene maps to chromosome 16 in 16q22.2-q22.3, in proximity to several loci associated with inherited craniofacial disease genes^3^. Second, human *Cfdp1* was shown to be a target of the TFII-I transcription factors, which are in turn encoded by genes suggested to be primary candidate genes responsible for craniofacial abnormalities and other defects associated with the Williams-Beuren disease^4^. Third, mouse *Cfdp1*, also called CP27, is up-regulated during experimentally induced osteodifferentiation in mouse and is therefore proposed to be required for bone differentiation^5^. Fourth, cleft palate induced in mouse by persistent expression of PAX 3 gene in neural crest correlated with *Cfdp1* down-regulation^6^. Finally, in *zebrafish*, the CFDP1 protein, also called RLTPR, is expressed in the branchial arches^7^ which lead to the craniofacial structures.

Recent findings on the *Drosophila* YETI protein indeed provided the first experimental evidence showing that members of the BCNT family can be essential for proper development and individual viability^8^. Moreover, functional studies suggest that in different organisms the BCNT proteins play a conserved role in chromatin regulation. In particular, yeast SWC5, *D. melanogaster* YETI and human CFDP1 belong to related ATP-dependent chromatin remodeling complexes of INO-80 family that share evolutionary conserved subunits and catalyze the exchange of histone variant H2A.V with the canonical H2A^8^,^9^,^10^.

Experimental evidence accumulated in the last decade showed that mutations in genes encoding proteins governing chromatin architecture and function can affect dramatically cell differentiation and cause complex human developmental disorders^11^,^12^,^13^,^14^. It is then clear that, the characterization of chromatin factors controlling cell differentiation represents a foothold for the comprehension of mechanisms underlying the onset of human developmental diseases.

Here, using a multidisciplinary experimental approach, we studied the effects of human *Cfdp1* transgene expression in *Drosophila melanogaster*. We found that when expressed in wild-type flies, CFDP1 protein binds to chromatin and behaves as a dominant-negative leading to diverse phenotypic defects similar to those produced by the lack of endogenous YETI^8^. In addition, using GST-pull down assays, we found that both YETI and CFDP1 undergo self-interactions and can physically interact each other. These results strongly suggest that both proteins are able to form homodimers *in vivo* and that, when simultaneously expressed in the same cells, can undergo heterodimerization which in turn would trigger the dominant negative effect observed in flies.

Overall, these findings are relevant in that they shed new light into unanticipated mechanistic clues of BCNT proteins and have impact on both basic and applied biomedical research.

## Results

### Ectopic expression of human *Cfdp1* transgene in *D. melanogaster* results in lethality and developmental defects

In order to investigate the effect of *Cfdp1* gene expression in flies, we constructed *D. melanogaster* transgenic lines carrying the *Cfdp1* human c-DNA under control of UAS regulatory sequences (see Material and Methods). Overexpression of the CFDP1 protein in *D. melanogaster* was performed by crossing two transgenic *UAS-Cfdp1* lines (15 and 16) to lines carrying ubiquitously active or tissue-specific GAL4 drivers. Western blotting analysis using a monoclonal antibody directed against CFDP1 was performed on protein extracts from human HeLa cells and from *w*, *elav-GAL4*^[*w*+]^*/w*; *UAS-Cfdp1*^[*w*+]^*/*+ larvae carrying the *UAS-Cfdp1* transgene under control of the *elev-GAL4* driver (Fig. 2; see Material and Methods for description of genetic crosses).

In HeLa cells, the anti-CFDP1 detected two sharp bands of about 50 kDa and 35 kDa (Fig. 2A), a result that is in accord with the existence of two isoforms of the protein produced by alternative splicing^1^. However, both bands have an apparently molecular weight higher than expected; in particular CFDP1 is 299 amino acids long and has a calculated molecular weight of 33.6 kDa. Such unusual mobility on SDS/PAGE was found to be due to an acidic stretch located at the N-terminal region of the protein^15^. In protein extracts from *w*, *elav-GAL4*^[*w*+]^*/w*; *UAS-Cfdp1*^[*w*+]^*/*+ female larvae, as espected the anti-CFDP1 recognizes only the band of about 50 kDa (Fig. 2A), produced by *UAS-Cfdp1* transgene expression; no staining is seen in *w/Y*; *UAS-Cfdp1*^[*w*+]^*/*+ control male larvae lacking the *elav-GAL4* driver (not shown).

Expression of *UAS-Cfdp1* transgenes in wild-type flies results in lethality and phenotypic abnormalities, depending on the driver used (Fig. 2B–D). With *GMR-GAL4*, an eye specific driver, ommatidia differentiation is affected (Fig. 2B), while with *Ms1096-GAL4* driver which is expressed throughout the wing pouch, a *vestigial*-like wing phenotype is produced (Fig. 2C). Expression of *UAS-Cfdp1* transgene driven by *tub-GAL4* or *elav-GAL4* induce strong lethality that mainly occurs at second/third instar larvae or pupal stages. The *tub-GAL4* driver is ubiquitously active, while *elav-GAL4*, was found to be expressed throughout the larval central nervous system and in peripheral sensory neurons as well as at all NMJs^16^. In addition, *elav-GAL4* driven expression of UAS transgenes was also detected in larval salivary gland cells^8^,^17^. Notably, *w,* *elav-GAL4*^[*w*+]^*/w*; *UAS-Cfdp1*^[*w*+]^*/*+ female larvae exhibited severely reduced brains and imaginal discs, with sporadic melanotic masses in their hemocoel (Fig. 2D) and are characterized by prolonged larval development. In addition, most of the pupae metamorphose and die at the adult pharate stage. Notably, about 10% of pharate adults show the failure of head eversion (Fig. 2D); only few escapers (about 1%) were found. Similar head eversion defects can results from abnormalities of muscular functions following *how*-*GAL4* driven expression in mesoderm and larval muscles of a transgene encoding a truncated form of Gemini3 motor protein^18^. We tested *UAS-Cfdp1* expression driven by *how*-*GAL4* and found strong pupal lethality and head eversion defects (not shown). It is then possible that CFDP1 expression driven by *elav-GAL4* in nervous system also affect muscular functions which result in failure of head eversion.

Finally, we tested the ability of *UAS-Cfdp1* to rescue or mitigate the lethal phenotype of *Yeti* mutants by genetic crosses. Overexpression of *UAS-Cfdp1* transgene was induced by the *tub-GAL4* driver in *w, UAS-Cfdp1*^[*w*+]/Y^; *LP1/LP1*; *tub-GAL4*^[*w*+]^*/*+ individuals that are homozygous for *LP1*, a loss-of-function allele of the *Yeti* gene^8^. The results showed that expression of *UAS-Cfdp1* fails to rescue the lethality due to the lack of YETI protein.

### CFDP1 binding to polytene chromosomes results in altered chromatin organization

In a previous study, we demonstrated that higher order chromosome organization and deposition of both H2A.V and H2A are severely impaired in *Yeti* null mutants^8^. We then examined salivary gland polytene chromosome morphology and chromosomal levels of both H2A.V and H2A in *w,* *elav-GAL4*^[*w*+]^*/w*; *UAS-Cfdp1*^[*w*+]^*/*+ larvae and in *UAS-Cfdp1*^[*w*+]^*/UAS-Cfdp1*^[*w*+]^ control larvae lacking the *elav-GAL4* driver. We found that about 66% of salivary gland cells show highly disorganized polytene chromosome organization compared to those of the control strain (Fig. 3A). In addition, the results of immunostaining experiments clearly show strong decrease in H2A.V and H2A protein levels on polytene chromosomes of *w,* *elav-GAL4*^[*w*+]^*/w*; *UAS-Cfdp1*^[*w*+]^*/*+ female larvae, compared to the controls (Fig. 3B–E and Supplementary Fig. 1). Western blotting assays of whole protein extracts from larval salivary gland and brains also depicted a reduced overall amount of H2A.V and H2A proteins in *elav-GAL4*^[*w*+]^*/w*; *UAS-Cfdp1*^[*w*+]^*/*+ (Fig. 4A,B).

It is possible that strong decrease in H2A.V and H2A protein levels and impairment of chromosome organization are aberrant consequences due to the binding of the CFDP1 protein to chromatin in *Drosophila* cells. To test this hypothesis and to get further insight into the function of CFDP1, salivary gland polytene chromosomes of *w,* *elav-GAL4*^[*w*+]^*/w*; *UAS-Cfdp1*^[*w*+]^*/*+ female larvae were immunostained with anti-CFDP1 antibody. As shown in Fig. 3F, this antibody produces strong staining of polytene chromosomes with some appearance of banding, while no staining was observed on polytene chromosomes of either *w/Y*; *UAS-Cfdp1*^[*w*+]^*/*+ male or *UAS-Cfdp1*^[*w*+]^*/UAS-Cfdp1*^[*w*+]^ female larvae lacking the *elav-GAL4* driver (not shown).

Together, these data indicate that CFDP1 binding to chromatin, results in depletion of both H2A and H2A.V, which in turn may produce a cascade effect disrupting chromatin/chromosome organization.

### Simultaneous expression of *UAS-Cfdp1* and *UAS-Yeti-GFP* transgenes in *D. melanogaster*

Overall, the phenotypes of lethality, prolonged larval development, reduced brain and imaginal disc size and highly disorganized polytene chromosomes organization, together with decreased levels of H2A.V and H2A found in *w,* *elav-GAL4*^[*w*+]^*/w*; *UAS-Cfdp1*^[*w*+]^*/*+ larvae are highly reminiscent of the aberrant effects found in *Yeti* null mutants^8^. It is then conceivable that expression of the CFDP1 protein in flies induces a dominant-negative effect possibly disrupting the function of endogenous YETI, as reported for other human genes expressed in *Drosophila*^19^.

We demonstrated previously that YETI-GFP fusion protein binds to polytene chromosomes where it is recruited at DAPI-positive bands^8^. Here, we took advantage of YETI-GFP expression, to test whether the defects due to activation of *UAS-Cfdp1* could be due to depletion of endogenous YETI from chromatin. We performed sequential immunostaining of polytene chromosomes of larvae expressing *UAS-Cfdp1* and *UAS-Yeti-GFP* transgenes, to visualize simultaneously both CFDP1 and YETI-GFP. The results of this experiment show that YETI-GFP and CFDP1 proteins mainly colocalize on the same polytene chromosomes, with CFDP1 showing a slightly broader distribution (Fig. 5A,B). Most importantly, looking at polytene chromosome banding pattern of YETI-GFP in genetic background expressing YETI-GFP alone^8^ or both YETI-GFP and CFDP1 (Fig. 5A,B), it appears that the distribution of the fusion protein is unaffected, at least at cytological level.

Together, these results show that CFDP1 and YETI-GFP share a similar pattern of recruitment on polytene chromosomes and indicate that both lethality and aberrant phenotypes induced by CFDP1 expression in *D. melanogaster* are unlikely to be due to significant depletion of endogenous YETI protein from chromosomes.

### GST pull-down experiments

On the light of the above results, it is possible that aberrant effects produced by *UAS-Cfdp1* expression in flies may be due to the *in vivo* formation of YETI-CFDP1 inactive heterodimers. If this was true, we also expect that both proteins should be able to undergo physiologically self-dimerization.

To test these hypotheses, we performed GST pull-down experiments. Whole lysates from larvae expressing 3xFLAG::YETI protein under control of *elav-GAL4* driver, were incubated with GST alone, GST::YETI or GST::CFDP1 fusion proteins and analyzed by Western blot using anti-Flag antibody. As shown in Fig. 6A, both YETI and CFDP1 fusion protein, but not GST alone, specifically pulled down 3xFLAG::YETI. The interaction between CFDP1 and YETI was confirmed using GST::YETI to pull down CFDP1 protein from extracts of larvae expressing the *UAS-Cfdp1* transgene under control of *elav-GAL4* driver. As expected, the interaction of CFDP1 protein is apparent with GST::YETI, but not with GST alone (Fig. 6B).

Moreover, to test whether CFDP1 is able to undergo self-dimerization, we also performed GST pull-down assay with GST::CFDP1 using a lysate from HeLa cells transfected with pcDNA3.1/nV5-DEST::CFDP1. As shown in Fig. 6C, V5::CFDP1 fusion protein is detectable using anti-V5 antibody in samples pulled down by GST::CFDP1, but not by GST alone. Taken together, these findings indicate that both YETI and CFDP1 are able to undergo self-dimerization and that, when expressed simultaneously in the same cells, can form inactive YETI-CFDP1 heterodimers that bind chromatin.

Moreover, to map the portion of the YETI protein implicated in self-interactions, we tested the N-terminal or C-terminal portion of YETI in GST assays. Whole lysates from larvae expressing both 3xFLAG::YETI-N-ter and 3xFLAG::YETI-C-ter fusion proteins^8^, under control of *elav-GAL4* driver were incubated with GST alone or with GST::YETI fusion protein and analyzed by Western blot using anti-Flag antibody. As shown in Fig. 6D, GST::YETI specifically pull-down 3xFLAG::YETI-C-ter that corresponds to the highly conserved C-terminal BCNT domain^8^. This result provides evidence for a role of YETI BCNT domain in mediating homodimerization.

## Discussion

To investigate the function and mechanism of action of CFDP1 protein, we have studied the *in vivo* effects of its expression in *D. melanogaster*. We found that when overexpressed in wild-type flies, CFDP1 binds to chromatin and causes the following defects: disruption of polytene chromosome organization, decreased levels of H2A.V and H2A, reduction of brains and imaginal discs size, occurrence of melanotic masses, prolonged larval development and lethality (Figs 2, 3, 4 and Supplementary Fig. 1). It is worth noting that all these aberrant phenotypes, rather than being the result of a generalized disruption of development, have been observed in *Yeti* null mutants^8^. Then, it appears that the expression of CFDP1 in *D. melanogaster* is likely to disrupt the normal function of YETI and results in a dominant-negative effect.

What the molecular nature of such dominant negative effect may be? Three hypotheses can be considered. First, the aberrant phenotypes produced by CFDP1 expression may be explained in terms of competition between CFDP1 and YETI proteins for binding to chromatin targets, which may result in a significant depletion of YETI from chromatin. Second, YETI-CFDP1 heterodimers can efficiently be formed *in vivo*, but they are unable to bind chromatin, thus resulting in a depletion of YETI from chromatin. However, the observation that polytene chromosome distribution and levels of YETI-GFP are not significantly affected, at least at a cytological level, when simultaneously expressed together with CFDP1 (Fig. 5A,B), strongly argues against these two hypotheses. Third, the dominant negative effect may be due to *in vivo* formation of YETI-CFDP1 heterodimers still capable of binding to chromatin, but unable to establish further interactions with specific partners of YETI. It has indeed been reported that the expression of human genes in *D. melanogaster* is responsible for dominant negative effects, through titration of endogenous proteins by the human counterpart to form inactive heterodimers^16^.

The latter hypothesis gained experimental support by the results of our cytological and biochemical experiments. First, simultaneous immunolocalization of CFDP1 and YETI-GFP on polytene chromosome showed that these proteins mainly colocalizes at multiple chromosomal sites (Fig. 5). Second, GST pull-down assays provide evidence for physical interaction between CFDP1 and YETI (Fig. 6A,B).

Together, these findings converge in indicating that the dominant negative effect produced by CFDP1 expression is most likely due to the binding of YETI-CFDP1 heterodimers to chromatin. This would result in overall depletion of functional YETI protein and disruption of H2A/H2A.V exchange, similarly to what we found in *Yeti* null genetic background^8^. A consequent breakdown of chromatin organization would then occur, thus altering global gene expression and leading to phenotypic abnormalities similar to those produced by the lack of YETI protein^8^.

Moreover, GST pull-down assays indicated that both YETI and CFDP1 can undergo self-interactions (Fig. 6A,C), Notably, in the case of YETI, the C-terminal BCNT domain may be implicated in mediating self-interaction (Fig. 6D).

Although, we cannot completely rule out the possibility that a fraction of CFDP1 homodimers can form in *D. melanogaster* cells and bind separately to chromatin, it is unlikely that this would explain the occurrence of the observed dominant negative effect.

**Figure 1:**
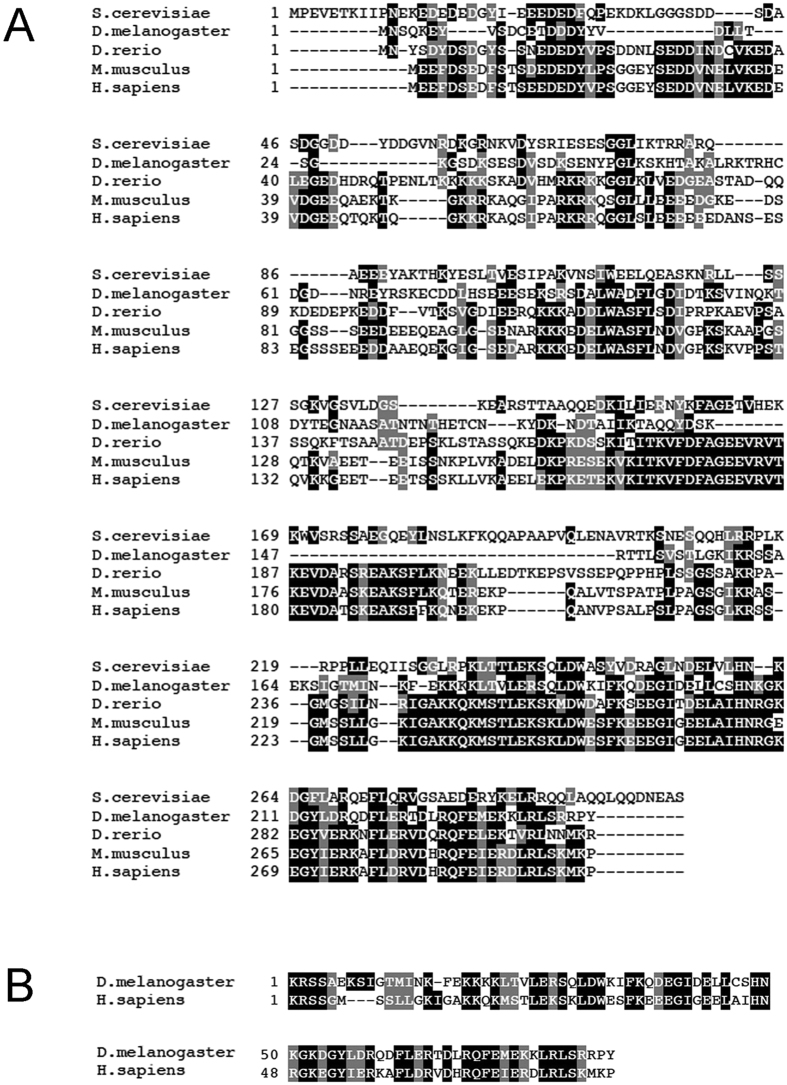
The BCNT protein family. (**A**) Blast alignments of BCNT protein sequences **f**rom *Saccharomyces cerevisiae, Danio rerio (zebrafish), Drosophila melanogaster, Mus musculus and Homo sapiens.* (**B**) The closest match between human CFDP1 and *Drosophila* YETI (50% identity) is found in the last 82 amino acids of the C-terminus and is called BCNT-domain.

**Figure 2:**
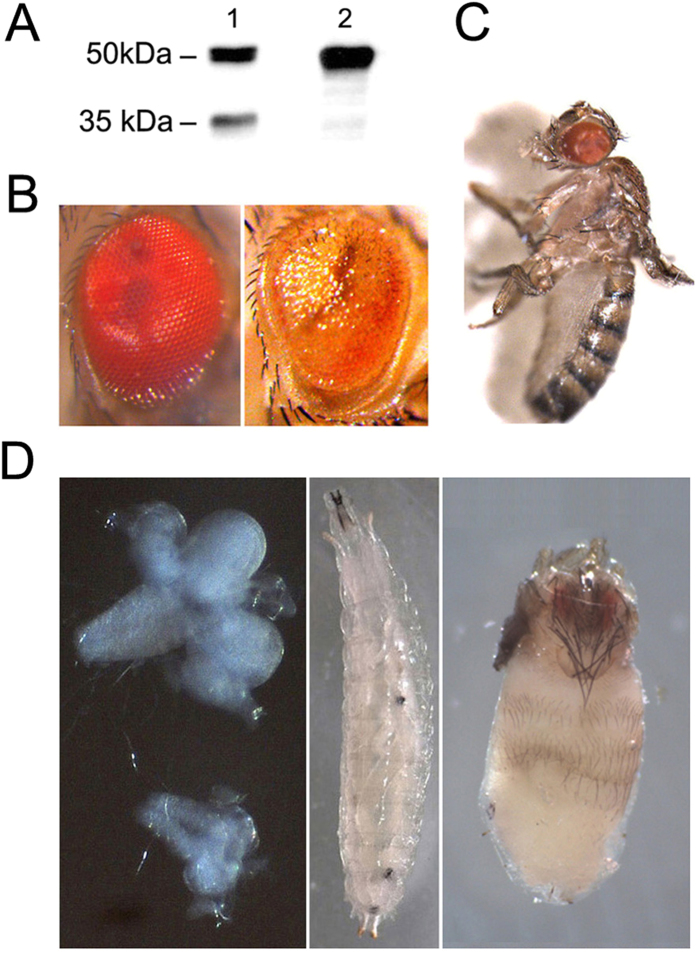
Expression of *UAS-Cfdp1* transgene activated by different *GAL-4* drivers. (**A**) Western blotting with anti-CFDP1 antibody of protein estracts from HeLa cells (lane 1) and from salivary glands and brains of *elav-GAL4; UAS-CFDP1* larvae (lane 2). Two bands of about 50 kDa and 35 kDa were detected by the anti-CFDP1 in Hela cells, while a single 50 kDa band was found in in *elav-GAL4*; *UAS-Cfdp1* larvae. (**B**) Overexpression of *UAS-Cfdp1* induced by *GMR-GAL4*; compared to the wild-type eye (left panel), the glazed-like phenotype consist in lack of ommatidia differentiation (left panel); the phenotype shows a 100% penetrance. (**C**) Overexpression of *UAS-Cfdp1* induced by the *MS1096-GAL4* driver causes strong reduction of wings portions. The phenotype shows a 100% penetrance. (**D**) Normal sized brain and imaginal discs of larvae carrying the *UAS-Cfdp1* trangene that is not activated (left panel, upper part); severely reduced brain and imaginal discs of larvae expressing *UAS-Cfdp1* (left panel, lower part); 60/266 (22%) dying larvae also develop sporadic melanotic masses in their hemocoel (middle panel), while 15/140 (10%) pharate adults show the failure of head eversion (right panel). Among 140 pupae only two vital adults (about 1%) were found. No effects were seen in the *GAL4* driver control flies lacking *UAS-Cfdp1* transgene.

**Figure 3:**
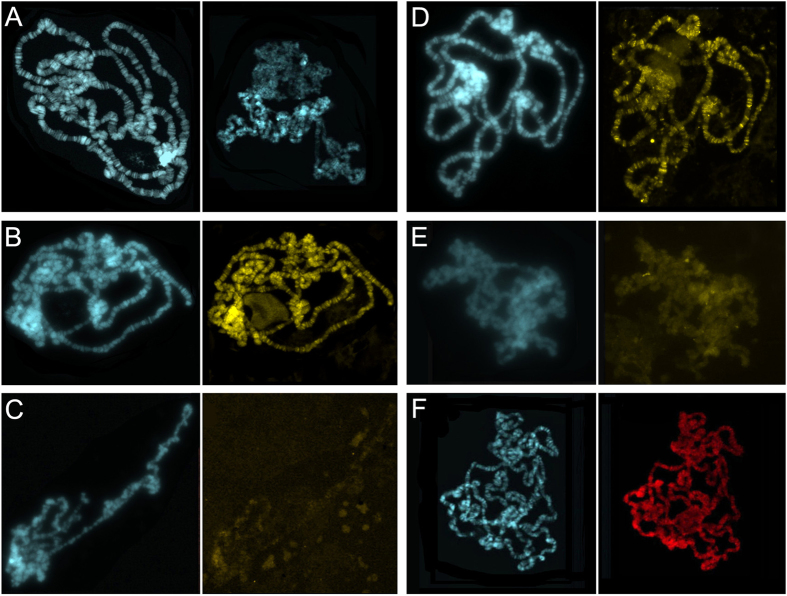
Chromatin defects in *elav-GAL4*^[*w*+]^*/w*; *UAS-Cfdp1*^[*w*+]^*/*+ larvae. (**A**) Comparison between polytene chromosomes of wild-type Oregon-R (left panel) and *w,* *elav-GAL4*^[*w*+]^*/w*; *UAS-Cfdp1*^[*w*+]^*/*+ (right panel) larvae stained with DAPI. Chromosome structure is highly disorganised in larvae expressing *UAS-Cfdp1*. Chromosome morphology was strongly affect in 231/350 (66%) figures scored were scored from three different replicates of the same cross. (**B**) Polytene chromosomes of *UAS-Cfdp1*^[*w*+]^*/UAS-Cfdp1*^[*w*+]^ larvae which do not express CFDP1 protein stained with DAPI (left panels); multiple H2A.V signals are present (right panel); (**C**) Polytene chromosomes of *w,* *elav-GAL4*^[*w*+]^*/w*; *UAS-Cfdp1*^[*w*+]^*/*+ larvae (left panel); H2A.V protein levels are decreased (right panel); (**D**) Polytene chromosomes of *UAS-Cfdp1*^[*w*+]^*/UAS-Cfdp1*^[*w*+]^ larvae stained with DAPI (left panels); multiple H2A signals are present (right panel); (**E**) Polytene chromosomes of *w,* *elav-GAL4*^[*w*+]^*/w*; *UAS-Cfdp1*^[*w*+]^*/*+ larvae (left panel); H2A protein levels are decreased (right panel); (**F**) Polytene chromosomes of *w,* *elav-GAL4*^[*w*+]^*/w*; *UAS-Cfdp1*^[*w*+]^*/*+ larvae stained with DAPI (left panel); strong staining with anti-CFDP1 antibody (right panel); in this case chromosome morphology is not dramatically affected.

**Figure 4:**
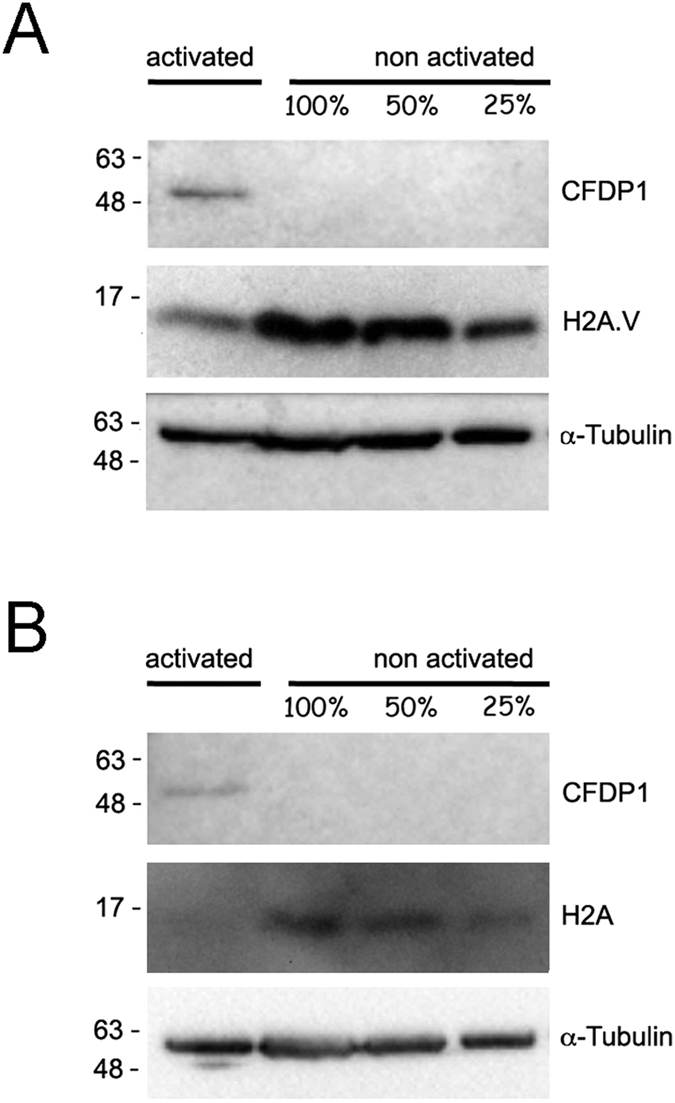
Decrease of total amount of H2A.V and H2A from protein extracts. Western blot showing the overall amount of H2A.V (**A**) and H2A (**B**) in *elav-GAL4*^[*w*+]^*/w*; *UAS-Cfdp1*^[*w*+]^*/*+ larvae expressing CFDP1 protein (activated) and in *UAS-Cfdp1*^[*w*+]^*/UAS-Cfdp1*^[*w*+]^ control larvae which do not express CFDP1 (non activated). 50% and 25% dilutions of protein extracts were also loaded. The α -tubulin is used as loading control. A significant decrease in the total amount of both H2A.V and H2A is evident in activated larvae compared to non activated controls.

**Figure 5:**
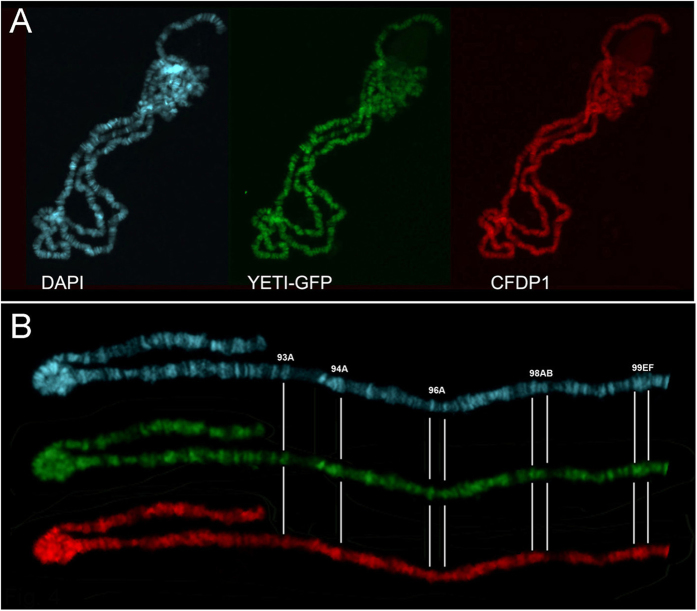
CFDP1 colocalizes with and YETI-GFP on polytene chromosome of *w,* *elav-GAL4*^[*w*+]^*/w; UAS-Yeti GFP*^[*w*+]^*/UAS-Cfdp1*^[*w*+]^ larvae. Polytene chromosomes from *w,* *elav-GAL4*^[*w*+]^*/w; Yeti-GFP*^[*w*+]^*/UAS-Cfdp1*^[*w*+]^ female larval progeny were stained with DAPI (blue) and immunostained with both anti-GFP (green) and anti-CFDP1 (red) antibodies. (**A**,**B**) YETI-GFP is recruited at DAPI-positive bands; the same patter has been observed when YETI-GFP is expressed in absence of CFDP1^8^; YETI-GFP and CFDP1 proteins mainly colocalize, with CFDP1 showing a slightly broader distribution. (**B**) A detail of 3R chromosome clearly showing the recruitment of YETI-GFP at DAPI-positive bands. Polytene chromosome bands 93A, 94, 96A, 98B and 99EF are shown as landmarks.

**Figure 6:**
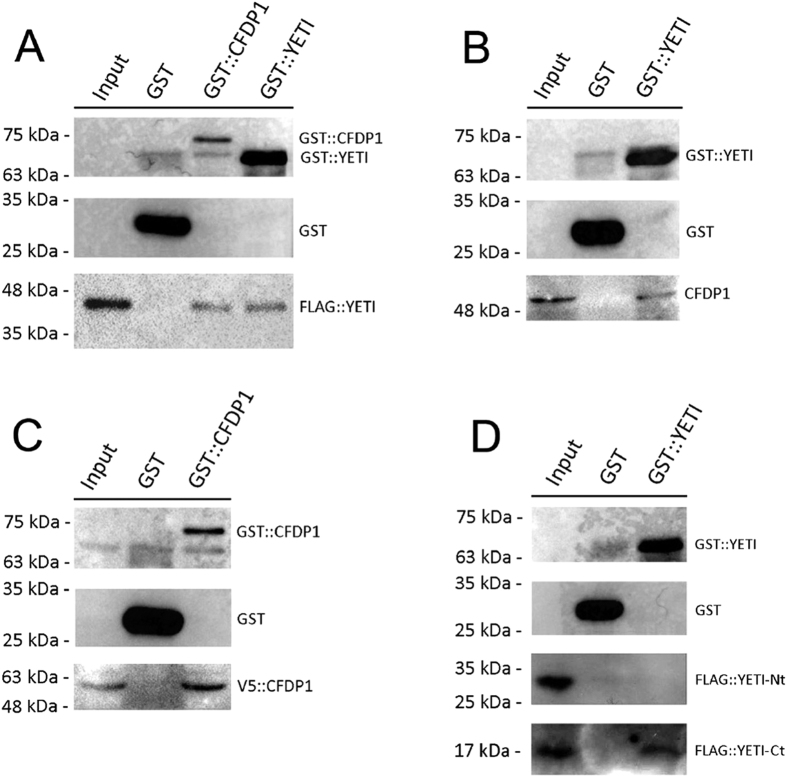
GST-pull down assayes. (**A**) GST-pull down experiments were performed with the indicated GST, GST::YETI and GST::CFDP1 fusion proteins, which were incubated with whole Drosophila larvae lysates expressing FLAG::YETI. (**B**) GST-pull-down assay with GST, GST::YETI, and whole larvae extracts expressing CFDP1 protein. (**C**) GST pull-down assay with GST (control) and GST::CFDP1 (Pull-down) of mammalian HeLa cells extracts expressing V5::CFDP1. (**D**) GST-pull-down assay with GST, GST::YETI, and whole larvae extracts expressing 3xFLAG::YETI-N-ter or 3xFLAG::YETI-C-ter proteins. GST::YETI pulled down 3xFLAG::YETI-C-ter, but not 3xFLAG::YETI-N-ter. Bound proteins were analyzed by SDS–PAGE, followed by western blotting with antibodies against the FLAG-tag (**A,D**), CFDP1 (**B**) and V5-Tag (**C**). Total protein extracts were used as control (input). Two GST-pull down replicates were performed.

The human CFDP1 and *Drosophila* YETI proteins share a similar function in chromatin remodeling^1^,^2^. However, we found that the expression of *UAS-Yeti* rescues the lethality of *Yeti* null mutants, while that of *UAS*-*Cfdp1* does not^8^. How to explain this result? It is important to point out that at the amino acid level the highest identity between the two proteins (about 50%) is found in the evolutionarily conserved BCNT domain^1^,^2^ (Fig. 1B), that is likely to play a functional role. Our data indeed suggest that dimerization is a conserved functional trait of BCNT domain. On the light of these results, the inability of human CFDP1 to rescue the lack of YETI in *D. melanogaster* can be interpreted as due to a lower conservation between the two proteins found in the remaining portion of their amino acid sequence (Fig. 1A,B).

Several chromatin proteins are known to undergo homodimerization *in vivo* through coiled coil interactions^20^,^21^,^22^,^23^,^24^,^25^,^26^. Interestingly, the secondary structure analysis of both CFDP1 and YETI predicts coiled-coil α -helix stretches located at their C-terminal portion corresponding to the BCNT domain (Supplementary Fig. 2).

**Figure 7:**
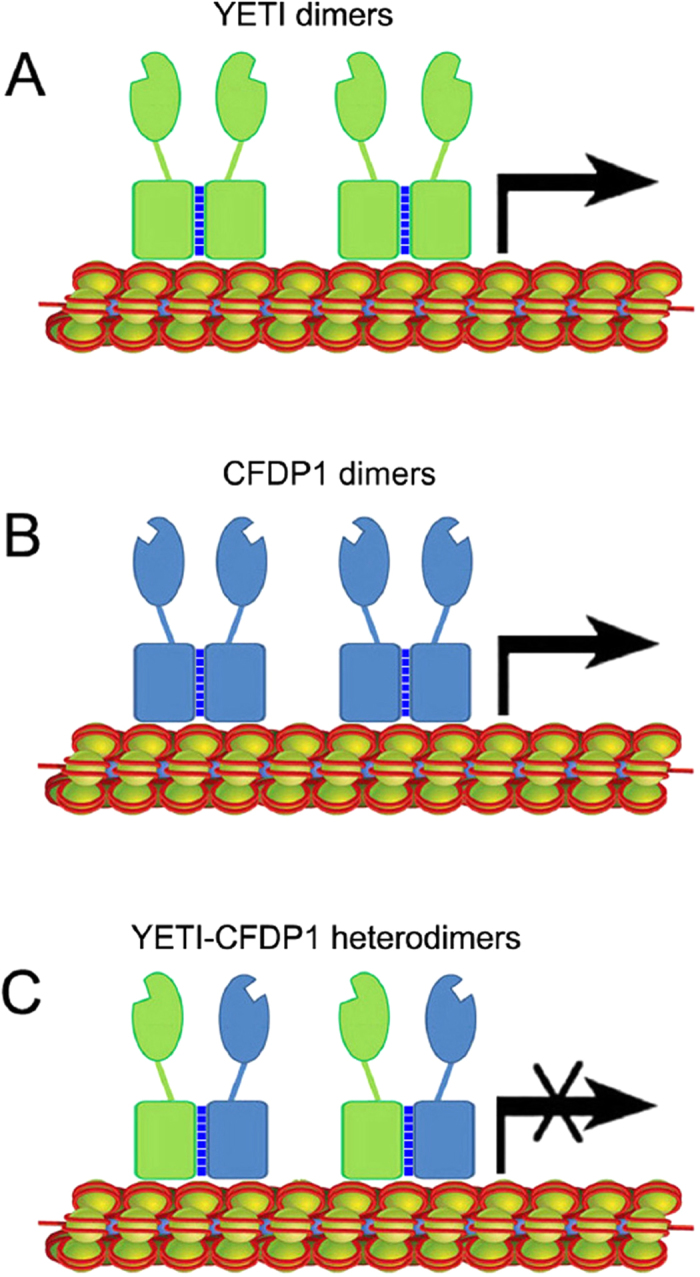
Model for homodimerization of YETI and CFDP1 and heterodimer formation. (**A**,**B**) Self-association of YETI and CFDP1 to form functional dimers in *Drosophila* and human cells, respectively. (**C**) Formation of YETI-CFDP1 inactive heterodimers capable of binding to chromatin, but unable to establish further interactions with specific partners of YETI. Dark-blue dashed lines indicate the interactions between C-terminal BCNT domains.

These findings, together with the results of our previous study showing that the BCNT domain of YETI is required for binding to polytene chromosomes^8^, suggest that homodimerization is also necessary to promote the chromatin binding ability of YETI and more in general of BCNT proteins. A scheme summarizing our model of homodimer and heterodimer formation is shown in Fig. 7.

In conclusion, by combining genetics, cytological and biochemical approaches, we shed new light on the mechanism of action of YETI and CFDP1 proteins. In particular, we provide the first experimental evidence suggesting that BCNT proteins can undergo *in vivo* homodimerization mediated by the evolutionarily conserved BCNT domain. These results are also relevant to biomedical research, since mutations of *Cfdp1* gene has been implicated in the onset of craniofacial anomalies that represent one of the main cause of infant mortality and disability in humans.

## Methods

### Drosophila strains and genetic crosses

Fly cultures and crosses were carried out at 25 °C in standard cornmeal yeast medium. The GAL4-driver lines used in this work were obtained from the Bloomington Drosophila Stock Center. Overexpression the human CFDP1 protein in *D. melanogaster* was induced in the offspring of crosses between transgenic *w/w; UAS-Cfdp1*^[*w*+]^*/UAS-Cfdp1*^[*w*+]^ homozygous females and males of different GAL4 drivers. For example, to induce CFDP1 expression in salivary glands cells, *w/w; UAS-Cfdp1*^[*w*+]^*/UAS-Cfdp1*^[*w*+]^ homozygous females were crossed to *w*, *elav-GAL4*^[*w*+]^*/Y;* + */*+ males to obtain *w*, *elav-GAL4*^[*w*+]^*/w*; *UAS-Cfdp1*^[*w*+]^*/*+ larval female progeny. Among the male offspring of these crosses, the control is represented by *w/Y; UAS-Cfdp1*^[*w*+]^*/*+ males lacking the *elav-GAL4* driver. Overexpression of CFDP1 was induced in both wild-type and *Yeti* mutant backgrounds. Simultaneous expression of *YETI-GFP* and *CFDP1* proteins was obtained by crossing *w*, *elav-GAL4*^[*w*+]^*/w*, *elav-GAL4*^[*w*+]^*/UAS-Yeti-GFP/Yeti-GFP* females to *w/Y; UAS-Cfdp1*^[*w*+]^*/UAS-Cfdp1*^[*w*+]^ males. The full-length YETI amino acid sequence is 241 amino acids in lenght; 3XFLAG::YETI-Nter corrisponds to residues 1–166, while 3xFLAG::YETI-C-ter corresponds to 145–241. The BCNT domain corresponds to to residues 159–238. A detailed description of these lines can be found in ref. 8.

### Generation of *UAS-Cfdp1* transgenic lines

*D. melanogaster* carrying the *UAS-Cfdp1* transgene were generated as follows: The full ORF of *Cfdp1* gene was introduced downstream of the UAS regulatory sequences of the pUAST transformation vector. The pUAST-*Cfdp1* construct was injected in *D. melanogaster* pre-blastoderm embryos of genotype *w*^*1118*^ and transgenic flies were selected using the *white*^+^ phenotypic marker. We isolated and balanced three lines called, 3, 15 and 16. Line 3 was mapped to the X chromosome, while line 15 and 16 were mapped to chromosomes 2 and 3, respectively.

### Cytology and immunostaining

Immunolocalization experiments to *Drosophila* polytene chromosomes were according to Deuring *et al.* 25. Anti-GFP rabbit polyclonal antibodies was used at 1:100 dilution, while anti-CFDP1 mouse monocolonal antibodies (Abnova, H00010428-M4, clone 5B7) at 1:50. Anti-H2A.V or anti-H2A (Millipore) antibodies were used at 1:100 dilution. After three washes in PBS, they were incubated with secondary antibodies Alexa Fluor 555 goat anti-rabbit IgG (1:300) for 1 h at room temperature and DAPI were used to DNA staining. Polytene chromosome preparations were analyzed by using a computer-controlled Nikon Eclipse 50i epifluorescence microscope equipped with a CCD camera. Measurements of polytene chromosome fluorescence levels of H2A.V and H2A were performed using the ImageJ software. For each histone, 25 chromosome figures were analysed in *w*, *elav-GAL4*^[*w*+]^*/w; UAS-Cfdp1*^[*w*+]^*/*+ larvae and in *w/w UAS-Cfdp1*^[*w*+]^*/UAS-Cfdp1*^[*w*+]^ control larvae.

### Cloning and preparation of DNA constructs

Full-length cDNAs of *Yeti* and *Cfdp1* were cloned into a pDONR-221 (Invitrogen) and then subcloned into pDEST-15 (Invitrogen) bacterial expression vector carrying Glutathione S-Transferase (GST) tag N-terminally. *Cfdp1* cDNA was also transferred into pcDNA3.1/nV5-DEST vector (Invitrogen) for transient expression of V5::CFDP1 protein in HeLa cells.

### Production and precipitation of GST fusion proteins

Recombinant GST::YETI and GST::CFDP1 were produced in BL21 (DE3) pLysS competent cells. Single clones expressing each of the individual GST fusion plasmids were grown in 3 ml of broth medium (LB) with ampicillin (100 g/ml) at 37 °C. Overnight cultures were diluted 1:20 with fresh LB with ampicillin, and grown at 37 °C to an A600 of 0.6–0.8 with vigorous agitation. Protein expression was induced by 1 mM isopropyl-beta-D-galactopyranoside (Sigma–Aldrich) at 37 °C for 4 h. GST fusion proteins were purified on 50 μ l of glutathione-coated agarose beads (Sigma–Aldrich) 1 h at 4 °C, by using 500 μ l of bacterial lysate (GST alone, GST::YETI and GST::CFDP1). Beads were washed three times with 1 ml of PBS plus protease inhibitor cocktail (Roche) and stored in the same buffer. Production of proteins of the predicted molecular mass was verified by Western blotting using antibodies to GST (1:1000, Abgent).

### GST Pull-Down assays

GST Pull-Down assays were performed by using third-instar Drosophila larvae expressing 3xFLAG::YETI or CFDP1 under control of elav-Gal4 driver, and HeLa cells transiently transfected with pcDNA3.1/nV5-DEST::CFDP1 plasmid. Whole larvae/cell extracts were prepared in GST pull-down lysis buffer (20 mM Tris, pH 8, 150 mM NaCl, 0.5% Nonidet P-40, 1 mM EDTA) plus protease inhibitor cocktail/1 mM PMSF, and then 50 l of glutathione-agarose beads with bound GST, GST::YETI, or GST::CFDP1 were added. After overnight incubation with rotation at 4 °C, the supernatant was removed and the beads were washed five times with GST pull-down lysis buffer plus protease inhibitor cocktail. Co-precipitated proteins were eluted into sample buffer, separated by sodium dodecyl sulphate polyacrylamide gel electrophoresis (SDS–PAGE) and analysed by Western blotting.

### Western blot analysis

*D. melanogaster* protein extracts were prepared in sample buffer from salivary glands and brains. HeLa cells were lysated in sample buffer at 10.000 cells/μ l. All the samples were load in a polyacrylamide gel, transferred onto Polyvinylidene fluoride PVDF membrane and probed with different antibodies: rabbit anti-H2A.V (1:2500), rabbit anti-H2A (1:2500, Millipore), mouse anti-α -Tubulin (1:10000, Sigma), mouse anti-CFDP1 (1:500, Sigma-Aldrich, WH0010428M4, clone 5B7); rabbit anti-GST (1:1000, Abgent, AP1298a); mouse anti-Flag (1:1000, Sigma-Aldrich, F1804, clone M2); mouse anti-V5 (1:1000, Thermo Scientific, R960-25). The bands were immunodetected using the ECL kit from Thermo Scientific.

### Prediction of secondary protein structure

Prediction of α -helix stretches in the secondary structure of BCNT domain was performed using the Phyre2 algorithm (http://www.sbg.bio.ic.ac.uk/phyre2/html/page.cgi?id= index).

## Additional Information

**How to cite this article**: Messina, G. *et al.* Expression of human *Cfdp1* gene in *Drosophila* reveals new insights into the function of the evolutionarily conserved BCNT protein family. *Sci. Rep.* **6**, 25511; doi: 10.1038/srep25511 (2016).

## Supplementary Material

Supplementary Information
