# Supplementary material for: Expression of human Cfdp1 gene in Drosophila reveals new insights into the function of the evolutionarily conserved BCNT protein family
Source: Sci Rep. 2016 May 6;6:25511. doi: 10.1038/srep25511 (PMC4858687; doi:10.1038/srep25511)
Supplement: Supplementary Information [file srep25511-s1.pdf]

Supplementary figures

Expression of human *Cfdp1* gene in *Drosophila* reveals new insights into the function of the evolutionarily conserved BCNT protein family

Giovanni Messina, Maria Teresa Atterrato, Laura Fanti, Ennio Giordano and

Patrizio Dimitri

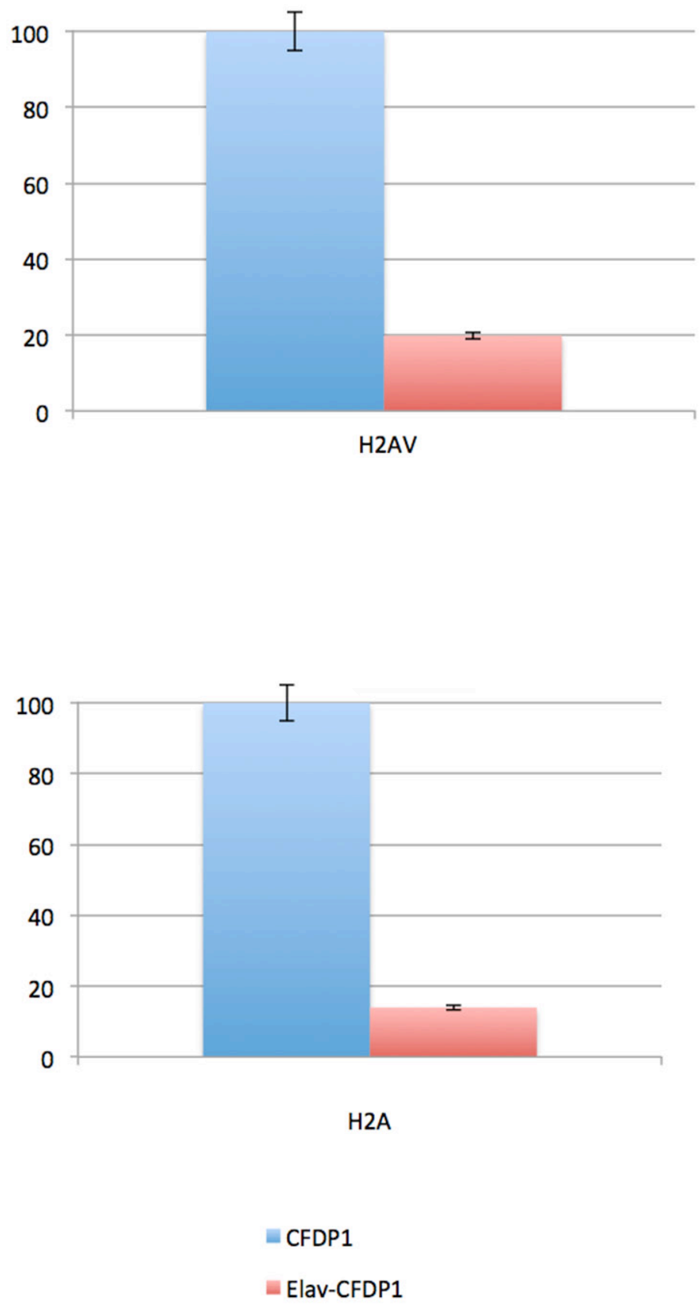

Supplementary Figure 1 Measurements of fluorescence levels of H2A.V and H2A on polytene chromosomes

About 80 and 85% decrease in levels of H2A.V and H2A, respectively, occurs in *elav-GAL4*$^{[w+]}$*/w;UAS-Cfdp1*$^{[w+]}$*/+* compared to *UAS-Cfdp1*$^{[w+]}$*/UAS-Cfdp1*$^{[w+]}$ controls. Measurements of polytene chromosome fluorescence levels of H2A.V and H2A were performed using the ImageJ software.

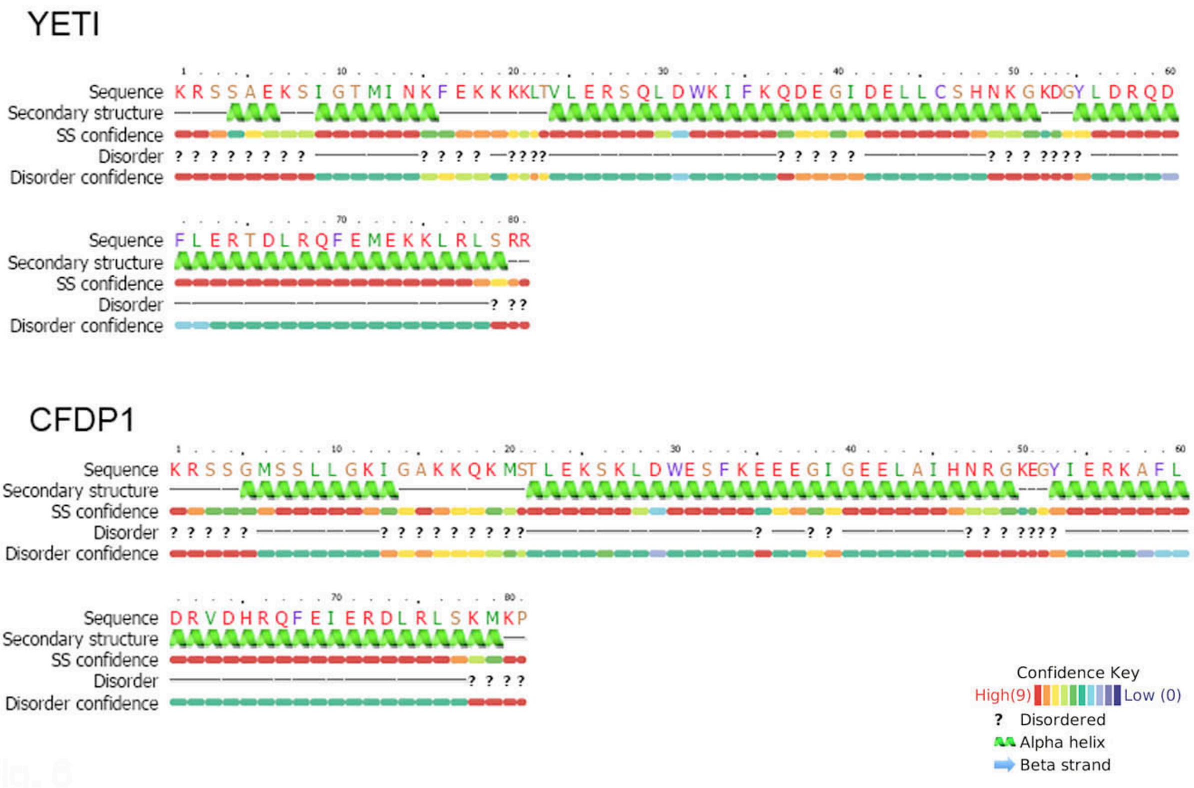

Supplementary Figure 2 Secondary structure analysis of BCNT domain between YETI and CFDP1

The secondary structure analysis of YETI and CFDP1, performed using the Phyre2 algorithm, predict α-helix stretches in the C-terminal BCNT domain of YETI (residues 181-237) and CFDP1. The α-helical coiled coil is the simplest of all protein-protein interaction motifs and consists of two or more α-helices that wrap around each other with a super-helical twist.
